# Efficient 2-phosphoglycolate degradation is required to maintain carbon assimilation and allocation in the C_4_ plant *Flaveria bidentis*

**DOI:** 10.1093/jxb/ery370

**Published:** 2018-10-23

**Authors:** Myles Levey, Stefan Timm, Tabea Mettler-Altmann, Gian Luca Borghi, Maria Koczor, Stéphanie Arrivault, Andreas PM Weber, Hermann Bauwe, Udo Gowik, Peter Westhoff

**Affiliations:** 1 Institute of Plant Molecular and Developmental Biology, Cluster of Excellence on Plant Sciences (CEPLAS), Heinrich-Heine-University, Universitätsstraße, Düsseldorf, Germany; 2 University of Rostock, Plant Physiology Department, Albert-Einstein-Straße, Rostock, Germany; 3 Institute of Plant Biochemistry and Cluster of Excellence on Plant Sciences (CEPLAS) Plant Metabolism and Metabolomics Laboratory, Heinrich Heine University, Universitätsstraße, Düsseldorf, Germany; 4 Max Planck Institute of Molecular Plant Physiology, Am Mühlenberg, Golm, Germany

**Keywords:** 2-phosphoglycolate phosphatase, C_4_ photosynthesis, Calvin-Benson cycle, *Flaveria bidentis*, photorespiration, RNAi suppression, transgenic lines

## Abstract

Photorespiration is indispensable for oxygenic photosynthesis since it detoxifies and recycles 2-phosphoglycolate (2PG), which is the primary oxygenation product of Rubisco. However, C_4_ plant species typically display very low rates of photorespiration due to their efficient biochemical carbon-concentrating mechanism. Thus, the broader relevance of photorespiration in these organisms remains unclear. In this study, we assessed the importance of a functional photorespiratory pathway in the C_4_ plant *Flaveria bidentis* using knockdown of the first enzymatic step, namely 2PG phosphatase (PGLP). The isolated RNAi lines showed strongly reduced amounts of PGLP protein, but distinct signs of the photorespiratory phenotype only emerged below 5% residual PGLP protein. Lines with this characteristic were stunted in growth, had strongly increased 2PG content, exhibited accelerated leaf senescence, and accumulated high amounts of branched-chain and aromatic amino acids, which are both characteristics of incipient carbon starvation. Oxygen-dependent gas-exchange measurements consistently suggested the cumulative impairment of ribulose-1,5-bisphosphate regeneration with increased photorespiratory pressure. Our results indicate that photorespiration is essential for maintaining high rates of C_4_ photosynthesis by preventing the 2PG-mediated inhibition of carbon utilization efficiency. However, considerably higher 2PG accumulation can be tolerated compared to equivalent lines of C_3_ plants due to the differential distribution of specific enzymatic steps between the mesophyll and bundle sheath cells.

## Teaser

Photorespiration is essential for maintaining high rates of C_4_ photosynthesis by preventing the 2-phosphoglycolate-mediated inhibition of carbon utilization efficiency.

## Introduction

Photosynthetic carbon assimilation is driven by ribulose-1,5-bisphosphate (RuBP) carboxylase (Rubisco) in all oxygenic phototrophs. The carboxylation of the primary acceptor RuBP leads to the formation of two 3-phosphoglycerate (3PGA) molecules that enter the Calvin–Benson cycle (CBC) to synthesize complex sugar compounds. However, in the presence of O_2_, RuBP oxygenation occurs continuously. This side-reaction results in the formation of only one 3PGA molecule and is accompanied by the production of one 2-phosphoglycolate (2PG) molecule. In contrast to 3PGA, 2PG cannot be directly used for carbon assimilation reactions (Bowes *et al.*, 1971; Lorimer, 1981; Ogren, 1984; Bauwe *et al.*, 2012). As well as the resulting excessive drain of previously fixed carbon from the CBC and the sequestration of P_i_, 2PG also strongly inhibits photosynthesis and prevents carbon allocation towards starch biosynthesis (Anderson, 1971; Kelly and Latzko, 1976; Wingler *et al.*, 2000; Flügel *et al.*, 2017).

To prevent extensive carbon losses, photorespiration evolved as a metabolic recycling system for 2PG, thus allowing high rates of photosynthesis in the presence of O_2_ (Bauwe *et al.*, 2012; Timm *et al.*, 2016). Re-incorporation of the carbon diverted into 2PG back into metabolism requires 2PG phosphatase (PGLP). Interestingly, a recent study has suggested that PGLPs originated in an Archaea-like ancestor, indicating that they first occurred before oxygenic photosynthesis evolved (Hagemann *et al.*, 2016). At that time, PGLP was involved in DNA repair mechanisms, which are physiological roles for which it has also been considered in heterotrophic plant tissues and other organisms (Winters *et al.*, 1994; Knight *et al.*, 2012; Hagemann *et al.*, 2016). In addition, phylogenetic analyses have shown that establishment of the full photorespiratory cycle was a co-evolutionary adaptation to oxygenic photosynthesis (Eisenhut *et al.*, 2008*b*; Hagemann *et al.*, 2013, 2016). However, carbon recovery by photorespiration is imperfect since only three out of the four carbon atoms present in two 2PG molecules are recycled to one 3PGA molecule and recirculated into the CBC. The fourth carbon atom is released as CO_2_ in the course of the photorespiratory glycine decarboxylase (GDC) reaction. In addition to CO_2_, GDC liberates ammonia, the refixation of which within the chloroplast consumes large amounts of ATP and reduced ferredoxin (Linka and Weber, 2005; Keys, 2006). Thus, it is not surprising that photorespiration represents an important target for genetic engineering approaches (Peterhänsel *et al.*, 2013; Betti *et al.*, 2016; Walker *et al.*, 2016), particularly to optimize the yield of C_3_ crops growing in environmental conditions that promote photorespiration (Ogren, 1984; Wingler *et al.*, 2000; Walker *et al.*, 2016; Simkin *et al.*, 2017).

It is considered that the GDC-mediated release of CO_2_ and NH_4_^+^ was the major driving force to suppress photorespiration during evolution. One physiological trait that resulted in very low rates of photorespiration was C_4_ photosynthesis, which independently evolved more than 60 times from C_3_ ancestors (Sage *et al.*, 2014; Sage, 2016). Whilst C_4_ plants are equipped with the full photorespiratory cycle (Zelitch *et al.*, 2009; Mallmann *et al.*, 2014), CO_2_ enrichment in the close vicinity of Rubisco diminishes RuBP oxygenation (Hatch, 1971; Ogren, 1984; Sage, 2004). Thus, CO_2_ is primarily fixed in the mesophyll using the O_2_-insensitive enzyme phosphoenolpyruvate carboxylase (PEPC), yielding oxaloacetate and, in turn, malate and aspartate. These organic acids diffuse into the bundle sheath cells where they are decarboxylated to release CO_2_ close to Rubisco, which exclusively localizes to this cell type in C_4_ plants (Hatch, 1987; Berry *et al.*, 2016). An additional advantage of strongly reduced RuBP oxygenation and, in turn, a lower carbon influx into photorespiration is a decline in the need for photorespiratory proteins. Thus, both the mRNA and protein levels of almost all photorespiration-related genes are reduced in C_4_ compared to C_3_ plants by a factor of three on average (Mallmann *et al.*, 2014). Despite the fact that strong reductions of photorespiration in organisms with carbon-concentration mechanisms imply that the whole process may be unnecessary, a broad range of studies have shown that the opposite is the case. The deletion of the photorespiratory genes in cyanobacteria, as well as in green and red algae, causes mutant phenotypes comparable to C_3_ plants (Suzuki *et al.*, 1989; Nakamura *et al.*, 2005; Eisenhut *et al.*, 2008*a*; Timm *et al.*, 2012; Rademacher *et al.*, 2016). Consistent with these results, Zelitch *et al.* (2009) reported that deletion of glycolate oxidase (GOX) also leads to a distinct photorespiratory phenotype in maize.

In this study, we generated transgenic *Flaveria bidentis* lines with decreased *PGLP* expression to examine the importance of photorespiration in C_4_ photosynthesis. This topic is of interest since our current knowledge on this type of photosynthesis is still restricted to maize (Zelitch *et al.*, 2009). In addition, we wanted to determine the mechanism by which C_4_ photosynthetic carbon assimilation adapts to changes in the internal 2PG content since it has been suggested to play a role as a signal molecule for environmental changes and in the regulatory interplay between photorespiration and other metabolic branches (Haimovich-Dayan *et al.*, 2015; Flügel *et al.*, 2017; Jiang *et al.*, 2018).

## Materials and methods

### Cloning of the *PGLP* RNAi construct and plant transformation

The total leaf mRNA was isolated from approximately 100 mg of leaf tissue (QIAprep RNeasy Kit, QIAGEN) and translated to cDNA (SMARTer RACE cDNA-Synthesis Kit, Clontech) using 1 µg of mRNA to generate the *Flaveria bidentis PGLP*-RNAi construct. Next, a 749 bp sub-fragment of the full-length cDNA (Supplementary Fig. S1A at *JXB* online) was amplified by PCR in the sense orientation using the oligonucleotide combinations *PGLP*-RNAi-sense-fw and *PGLP*-RNAi-sense-rev and in the antisense orientation via *PGLP*-RNAi-antisense-fw and *PGLP*-RNAi-antisense-rev (for sequences see Supplementary Table S1), and ligated into the pJET vector (ThermoFisher Scientific) for amplification and sequencing (LGC Genomics, Berlin, Germany). The sense arm was excised from pJET-PGLP(+) using the HindIII and XhoI restriction sites introduced and ligated into the pSK-Int vector (Guo *et al.*, 2003) to generate pSK-*PGLP*(+)-Int. Following amplification in *E. coli*, pSK-*PGLP*(+)-Int was digested using EcoRI and BamHI to introduce *PGLP*(–), previously excised from pJET-*PGLP*(–), to obtain pSK-*PGLP*(+)-Int-*PGLP*(–). Next, an adapter was constructed by mixing 50 ng of each synthetic oligonucleotides A-fw and B-rev, followed by incubation in water at 95 °C. After gradual cooling to room temperature (30 min), the adapter was digested with XhoI and ligated with the *PGLP*(+)-Int-PGLP(–) fragment, which was previously excised with XhoI and SacI from pSK-*PGLP*(+)-Int-*PGLP*(–). Subsequently, the adapter-*PGLP*(+)-Int-*PGLP*(–) fragment was cut with XmaI and ligated with the pBI121 vector (Jefferson *et al.*, 1987; Chen *et al.*, 2003), which was previously digested with XmaI and SacI, yielding the plasmid pBI121-*PGLP*-RNAi.

The constructed plasmid, pBI121-*PGLP*-RNAi, was checked via restriction analysis and sequencing prior to its transformation into *Agrobacterium tumefaciens* strain AGL1 (Lazo *et al.*, 1991) using electroporation. Following PCR verification of the recombinant *Agrobacterium* strains, pBI121-*PGLP*-RNAi was transformed into *F. bidentis* as previously described (Chitty *et al.*, 1994). Calluses resistant to kanamycin (200 µg ml^–1^) were selected and regenerated in normal (atmospheric CO_2_) air on SPM1 media supplemented with 3% sucrose. DNA was extracted from ~20 mg leaf tissue during cultivation of the putative transgenic plant lines, and the presence of the pBI121-*PGLP*-RNAi construct in the genome of *F. bidentis* was verified by the PCR amplification of a diagnostic fragment (1079 bp) using the oligonucleotides 35S-FW and *Act11*-Int-RV as indicated in Supplementary Fig. S1B. The PCR amplification was 1 min at 94 °C, 1 min at 60 °C, 1.5 min at 72°C; 35 cycles.

### Plant cultivation, propagation, and growth during CO_2_ transition

The continuous growth of eight validated transgenic *F. bidentis* lines (Supplementary Fig. S1C) was maintained by *in vitro* cultivation with regular transfers to new SPM1 media (Chitty *et al.*, 1994) at intervals of approximately 1 week. An initial test of the performance of the transgenic lines was conducted by transferring cuttings of the transformed calluses to soil and cultivating them in a growth cabinet under the following conditions: 16/8 h day/night at 25/22°C, 400–500 µmol m^−2^ s^−1^ light intensity ~60–70% relative humidity, and normal atmospheric air (0.039% CO_2_). Two transgenic lines were selected (L3 and L11), and their growth was characterized during a CO_2_-transition experiment, as follows. The plants were transferred from *in vitro* cultivation to soil/vermiculite (4:1 v/v) and grown under high CO_2_ (HC, 1% CO_2_) for 12 weeks in environmentally controlled conditions: (16/8 h day/night at 25/22 °C, ~130 µmol m^−2^ s^−1^ light intensity, and ~70% relative humidity (Percival Scientific). After conducting control experiments and sampling, the plants were transferred to normal air (low CO_2_, LC, 0.039% CO_2_) under otherwise equal conditions, and growth was monitored for at least 2 weeks, including sampling of leaf material on days 1 and 3. For seed production of L3 and, in particular, L11, continuous cultivation in HC was required (see Results). For comparison, we also examined the *in vitro* performance of a previously isolated Arabidopsis *PGLP1* mutant (*pglp1*; Schwarte and Bauwe, 2007). *pglp1* was grown next to the wild-type on half-strength media (Murashige and Skoog, 1962) with different sucrose concentrations (0, 1, and 2%) in either LC or HC. Plants were grown for 3 weeks under controlled conditions as described above, except that the temperature cycle was lowered to 20/18 °C day/night.

### Protein isolation and immunological studies

To isolate the total leaf protein, 100 mg of tissue was harvested after 8 h of illumination, ground to a fine powder in liquid nitrogen, and boiled for 4 min in protein isolation buffer (100 mM Tris-HCl, pH 7.8, 4 M urea, 5% SDS, 15% glycerol, and 10 mM 2-mercaptoethanol) as described by Heintz *et al.* (2006). Following centrifugation (15 000 *g*, 15 min), the protein concentration of the supernatant was quantified as described by Lowry *et al.* (1951) using an RC DC^TM^ protein assay kit (Bio-Rad). The extent of the reduction in PGLP protein in the transgenic lines was analysed by the separation of 60- or 10-µg leaf protein samples per genotype on a 12.5% SDS-PAGE gel (Schägger and von Jagow, 1987), followed by immunoblotting using standard protocols (Kyhse-Andersen, 1984). We used a specific antibody raised against the recombinant protein purified from Arabidopsis to estimate the abundance of PGLP (Flügel *et al.*, 2017), and signals of the transgenic lines were compared to dilution series of the wild-type extracts. Signals of the NADP-dependent malic enzyme (NADP-ME), Rubisco large subunit, histone H3, and glycine decarboxylase L-protein were used as loading controls (Mallmann *et al.*, 2014; Timm *et al.*, 2015). Protein–antibody complexes were visualized after incubation with a horseradish peroxidase-conjugated secondary antibody (Sigma-Aldrich) using a LAS-4000 mini-imager (GE Healthcare) according to the manufacturer’s instructions.

### Gas-exchange measurements

Leaf gas-exchange measurements were carried out using a LI-6400 system (LI-COR) in combination with a gas-mixing device (GMS600, (QCAL Messtechnik, Oberostendorf, Germany) to vary the oxygen concentration (5% or 40%, balanced with nitrogen). Measurements were taken within a 12-h period between 2 h after onset and 4 h prior to offset of the illumination from plants (*n*>4 per genotype) that had been grown for 12 weeks in HC. Measurements were taken on one leaf of the third leaf-pair, and the rate of net photosynthetic CO_2_ uptake (*A*) was recorded at 3-min intervals for 60 min in 5% O_2_. While keeping the leaf in the measurement chamber, the O_2_ concentration was first increased to 21% and then to 40%, and the measurements continued for 60 min in each case. All other parameters measured during the entire experiment were kept constant as follows: CO_2_ concentration, 400 ppm; block temperature, 25 °C; light intensity, 1000 µmol m^−2^ s^−1^; relative humidity, 60 to 70%; and flow rate, 300 µmol s^−1^. The oxygen inhibition of photosynthesis was calculated as described previously (Dai *et al.*, 1996).

### Determination of metabolite levels

Plants grown in HC for 12 weeks were used. Samples were taken after 8 h illumination at HC, and on day 1 and day 3 after the transfer to LC. At each time-point, the fourth leaf-pair was harvested by cutting the leaves from the plant directly into liquid nitrogen within the growth chamber. The material was then transferred into pre-cooled bags of aluminium foil and stored at –80 °C. Samples of 15–62 mg of ground leaf tissue were used for GC-MS analysis using the 7200 GC-qTOF system (Agilent). The extraction, derivatization, and analysis were carried out as described previously by Fiehn *et al.* (2000). The peak areas of each metabolite were determined using the MassHunter Quant software (Agilent) and normalized to the fresh weight, and the peak areas of the internal standard (ribitol), 2PG, glycerate, and 2-oxoglutarate were quantified using LC-MS/MS analysis from ~15 mg of ground leaf tissue as described by Arrivault *et al.* (2009, 2015).

### Statistical analysis

The data were examined using ANOVA and tested for significant differences using two-tailed Student’s *t*-tests (Microsoft Excel 10.0) or the Holm and Sidak test (Sigma Plot 11; Systat).

## Results

### Knockdown of *PGLP* expression in the C_4_ plant *Flaveria bidentis*

**Fig. 1.**
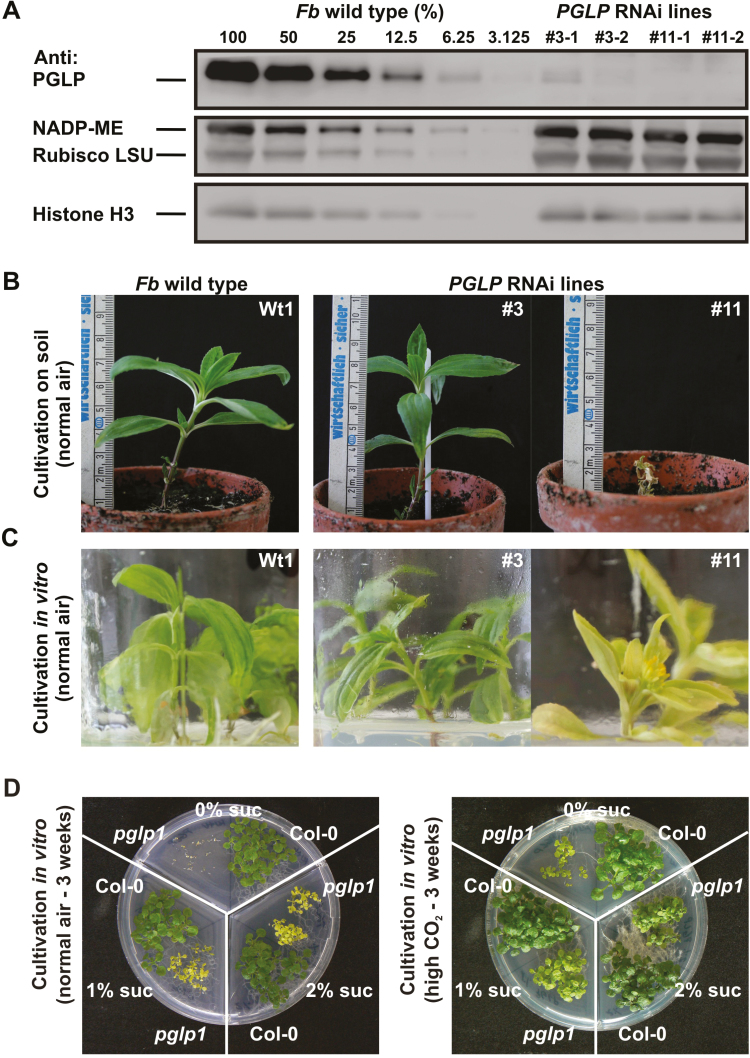
PGLP contents and phenotypes of the *PGLP*-RNAi lines. (A) Immunoblots of leaf protein extracts (60 µg) of the *Flaveria bidentis* (*Fb*) wild-type and *PGLP*-RNAi lines using a PGLP-specific antibody. Signals of the transgenic lines were compared to a dilution series of the wild-type protein extract to estimate the amounts of PGLP. Signals of the NADP-ME, Rubisco large sub-unit (LSU) and histone H3 were used as the loading controls. Similar results were obtained from three independent experiments. (B, C) Phenotypes of the wild-type and *PGLP*-RNAi lines L3 and L11 grown in low CO_2_ (atmospheric air) in soil for 1 week (B) and in *in vitro* cultivation (C). The growth of L3 on media was representative for all the other six validated *PGLP*-RNAi lines (Supplementary Fig. S1C). (D) Germination and growth of the Arabidopsis *pglp1* on media with or without sucrose supplementation(0, 1, and 2%) in low CO_2_ (atmospheric air, left) and high CO_2_ (1%, right) compared to the wild-type after 3 weeks.

An RNAi approach was undertaken to knockdown the expression of the photorespiratory *PGLP* in the C_4_ plant *F. bidentis* (Supplementary Fig. S1). Following transformation, eight transgenic calluses were obtained from *in vitro* cultivation on kanamycin- and sucrose-containing media in normal (atmospheric CO_2_) air, and the presence of the *PGLP*-RNAi construct was verified using PCR amplification of a diagnostic fragment (Supplementary Fig. S1C). We then examined the extent to which antisense repression of *PGLP* expression resulted in a reduction of the protein level in the primary transformants using a PGLP-specific antibody (Flügel *et al.*, 2017). From a dilution series using the wild-type leaf protein extract as a reference (Fig. 1A, Supplementary Fig. S2A), the abundance of PGLP in the transgenic lines were estimated: L23 (~100%) > L21 (~50%) > L9 (~20%) > L2 (~12.5%) > L18 and L20 (~10%) > L3 (~ 3–5%) > L11 (~0%).

### PGLP accumulates to below 3% of the wild-type which decreased *in vitro* growth and is lethal at ambient CO_2_ concentration

Cuttings of each line grown *in vitro* were prepared, planted in soil, and grown in LC (0.039% CO_2_) to propagate the transgenic lines for further investigation. All of the transgenic lines except for L11 could grow under these conditions (Fig. 1B; Supplementary Fig. S2B), and they flowered and produced fertile seeds. By contrast, growth of the L11 cuttings was quickly arrested after the transfer to soil, and they died within 1 week. A similar situation had already been observed during the *in vitro* cultivation of the transgenic lines: although L11 survived, its growth was considerably slower and was accompanied by a yellowish phenotype compared with all the other lines (Fig. 1C). It is notable that the growth properties of L11 resembled those observed with C_3_ Arabidopsis *pglp1*, a T-DNA insertional line that has previously been isolated (Schwarte and Bauwe, 2007; Flügel *et al.*, 2017). As shown in Fig. 1D, the germination of *pglp1* without external carbon was strongly reduced but gradually improved with sucrose supplementation (>1%). As expected, its growth was facilitated by cultivation with external carbon and elevated CO_2_ (1%), but it still did not fully recover to the level of performance exhibited by the wild type. Since several *F. bidentis* lines (e.g., L2, L9, L18, L20, and L21) exhibited strongly reduced abundance of PGLP protein (Supplementary Fig. S2A) but could grow and produce seeds in normal air, we initially tested the growth of the corresponding T1 generation. Kanamycin-resistant individuals were selected, transferred to soil, and grown in 1% CO_2_. As observed for L11, all of the lines examined were considerably stunted in growth compared to the wild-type, which clearly agreed with the stronger reduction in PGLP protein compared to the primary transformants (Supplementary Figs S2, S3).

### High-to-low CO_2_ transition reveals visual signs of the photorespiratory phenotype

**Fig. 2.**
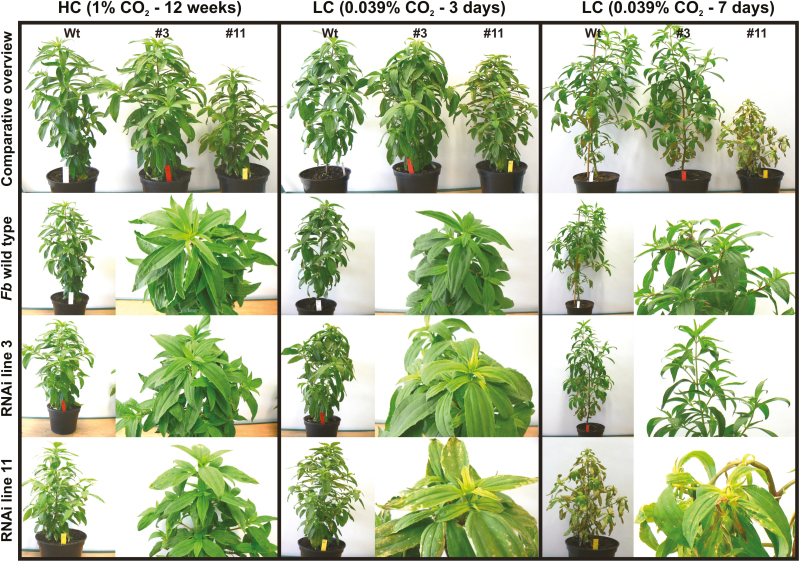
Phenotypes of *Flaveria bidentis* (*Fb*) *PGLP*-RNAi lines compared to the wild-type during transition from high to low CO_2_. Cuttings from the wild-type and the *PGLP*-RNAi lines L3 and L11 grown *in vitro* were transferred to soil and grown for 12 weeks in high CO_2_ (HC). The plants were subsequently transferred to low CO_2_ LC, and images were taken after 3 d and 7 d. Representative images for each genotype and for two independent experiments are shown.

Mutants defective in photorespiration are unable to grow in normal air, while growth like that of the wild-type can be achieved by cultivation in HC in many cases (Somerville, 2001; Zelitch *et al.*, 2009; Timm *et al.*, 2012; Timm and Bauwe, 2013). However, mutants initially grown in HC and transferred to normal air quickly become stunted in growth, and this is typically accompanied by severe stress symptoms in the leaves, such as bleaching and necrosis (Timm *et al.*, 2012; Pick *et al.*, 2013; Dellero *et al.*, 2015). To examine whether the *PGLP*-RNAi lines that we generated exhibit such features, we selected the two (L3, L11) with the lowest amounts of PGLP between the primary transformants and the wild-type for a CO_2_-transition experiment. Plants were initially grown in HC (1% CO_2_) for 12 weeks before being transferred to LC. In HC, L3 was visually indistinguishable from the wild-type whilst L11 also did not show alterations in leaf colour, but grew somewhat smaller (Fig. 2). After 3 d in LC, the leaves of both the RNAi lines showed bleaching, most distinctly in the developing sink leaves, and L11 (virtually PGLP-free) also developed necrotic lesions on its source leaves. After a further 4 d in LC, L3 recovered to some extent, and the plants grew similarly to the wild-type. In contrast, L11 had arrested growth and severe leaf necrosis, which eventually led to death 10–12 d after the transfer to air (not shown). Thus, *F. bidentis* tolerated a reduction in the abundance of PGLP to ~3–5% of wild-type levels before a photorespiratory phenotype was established.

### *PGLP*-RNAi lines accumulate very high levels of 2PG in atmospheric CO_2_ but exhibit minor changes in other photorespiratory intermediates

**Fig. 3.**
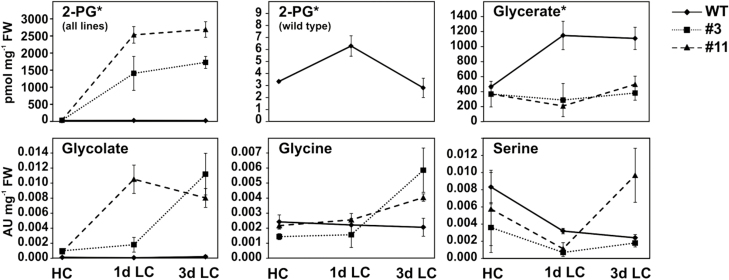
Accumulation of photorespiratory intermediates in leaves of *Flaveria bidentis PGLP*-RNAi lines L3 and L11 compared to the wild-type (WT) during transition from high to low CO_2_. Plants were grown for 12 weeks in high CO_2_ (HC, 1%) and then transferred to low CO_2_ (LC, atmospheric air) before being sampled at 1 d and 3 d. *Contents of metabolite marked with an asterisk were determined using LC-MS/MS (absolute amounts: pmol mg^−1^ FW); the others were determined using GC-MS (relative amounts: arbitrary units, AU mg^−1^ FW). Values are means (±SD) (*n*>3). For statistical evaluation, see Supplementary Datasheet S1.

In light of the phenotypic changes, we next examined to what extent reductions in PGLP affected the levels of 2PG and other metabolites of the photorespiratory pathway. Leaf material was harvested after 8 h of illumination in HC (suppressed photorespiration) and after 1 d and 3 d in LC (active photorespiration). The amounts of 2-phosphoglycerate and glycerate were quantified using LC-MS/MS (as absolute amounts, pmol mg^−1^ FW), while all other metabolites were quantified using GC-MS (as relative amounts, arbitrary units mg^−1^ FW). The wild-type had an absolute 2PG content of 3.45 ± 0.11 pmol mg^−1^ FW in HC, while both *PGLP*-RNAi lines exhibited slight, but not significant, increases in (11.43 ± 5.61 and 15.12 ± 5.33 for L3 and L11, respectively) (Fig. 3, Supplementary Datasheet S1). After the transfer to LC, 2PG transiently doubled in the wild-type at day 1 (6.51 ± 0.88) and returned to the HC level at day 3 (2.91 ± 0.83). In L3 (~3% PGLP), 2PG increased to 1384.68 ± 497.97 at day 1 and further increased to 1704.82 ± 176.70 at day 3. More dramatically, the accumulation of 2PG in L11 (virtually PGLP-free) was even greater and it had already increased to 2509.76 ± 242.42 after 1 d in LC, and remained at a similarly high level after 3 d (2666.56 ± 227.76) (Fig. 3).

By contrast, other pathway intermediates displayed less dramatic changes. In the wild-type, only glycerate reflected the induction of the photorespiratory pathway, increasing by ~2.5-fold at 1 d after the transfer to LC (from 467.35 ± 21.83 to 1150.78 ± 189.03 pmol mg^−1^ FW) and remaining at this level at 3 d (Fig. 3). This induction was absent in both the RNAi lines, in which the amount of glycerate remained at the HC level over the period examined. Glycolate did not show any significant changes during the CO_2_ transition in the wild-type but it increased between 10- and 14-fold after 3 d in LC in the RNAi lines (Fig. 3). The glycine levels showed a similar pattern, being unaffected in the wild-type and elevated by up to 4-fold after 3 d in LC in the transgenic lines. A less clear trend was observed for serine. In the wild-type, it decreased to ~30% of the HC level after transfer to LC. In comparison, both transgenic lines showed a slight but non-significant decrease in serine in HC, and the initial decrease at day 1 in LC was comparable to that observed in the wild-type. At day 3, L3 had a serine level similar to that of the wild-type, but in L11 it had increased by ~2-fold (Fig. 3). Overall, our analysis demonstrated that a reduction in PGLP abundance primarily caused a substantial accumulation of 2PG but no general disruption of photorespiration on the metabolic level.

### 2PG accumulation decreases the efficiency of C_4_ photosynthesis

**Fig. 4.**
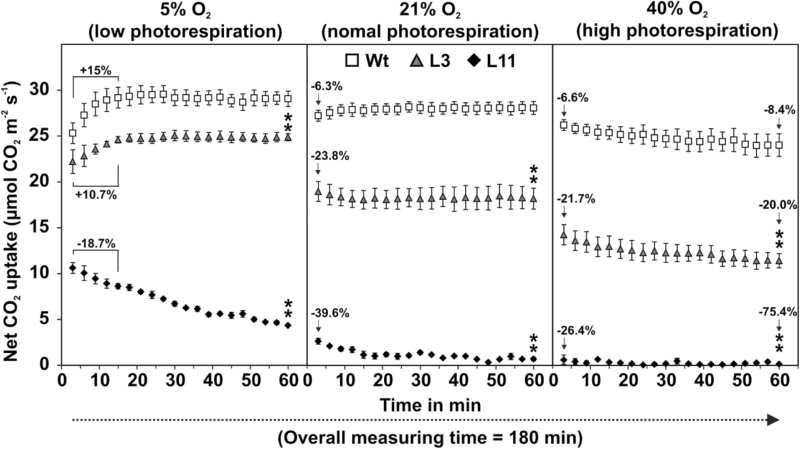
Photosynthetic rates of *Flaveria bidentis PGLP*-RNAi lines L3 and L11 compared to the wild-type (Wt) during stepwise changes in O_2_ concentration. Plants were grown for 12 weeks in high CO_2_ (1%) at atmospheric O_2_ concentration. Rates of photosynthetic net CO_2_ uptake (*A*) in fully expanded leaves were then recorded at 3-min intervals for 60 min in 5% O_2_, followed by 60 min in 21% O_2_, and 60 min in 40% O_2_. Values are means (±SE) from at least three biological replicates. Additional details of photosynthetic parameters are summarized in Table 1. Significant differences between the final value at a given O_2_ concentration and the initial value at that concentration were determined using Student’s *t*-test: **P<0.01. For statistical evaluation see Supplementary Datasheet S2.

It has been shown that 2PG is an efficient inhibitor of at least three enzymes involved in central carbon metabolism, namely phosphofructokinase (PFK), triosephosphate isomerase (TPI), and sedoheptulose-1,7-bisphosphatase (SBPase), and thus it has a major impact on photosynthetic ability (Anderson, 1971; Kelly and Latzko, 1976; Flügel *et al.*, 2017). We therefore conducted leaf gas-exchange measurements on *PGLP*-RNAi and wild-type plants that had been grown for 12 weeks in HC conditions. The plants were removed from the growth chamber, and the rates of photosynthetic net CO_2_ uptake (*A*) were continuously recorded for 60 min under low (5% O_2_), normal (21% O_2_), and high (40% O_2_) photorespiratory conditions. In the wild-type at 5% O_2_, following an initial increase of ~15%, values of *A* stabilized after 15 min (29.19 ± 1.12 µmol CO_2_ m^−2^ s^−1^) (Fig. 4). The *PGLP*-RNAi line L3 (~3% PGLP) also reached a stable value of *A* at this time-point, but the rate was significantly lower compared to that of the wild-type (24.60 ± 0.35). In addition, the initial increase in *A* was lower (~10.7%). Both the wild-type and L3 maintained stable *A* for the rest of the 1-h measurement period. In contrast, photosynthesis in L11 was strongly impaired from the beginning of the measurements and *A* had declined by ~18.7% after 15 min, and it further decreased to 4.34 ± 0.09 after 60 min (Fig. 4, Table 1). We did not observe any significant changes in stomatal conductance (*g*_*s*_) or transpiration rate (*Tr*) between the RNAi lines and the wild-type after 60 min in 5% O_2_. Notably, L11 showed an exceptionally high ratio of internal versus external CO_2_ concentration (*C*_i_/*C*_a_, Table 1).

**Table 1.** Gas exchange parameters of *PGLP*-RNAi lines compared to the wild-type

Parameter and O2 concentration	Wild-type	PGLP-RNAi L3	PGLP-RNAi L11
Net CO2 uptake, A (µmol CO2 m−2 s−1)			
5% O2	29.06 ± 0.84	24.88 ± 0.39**	4.34 ± 0.09**
21% O2	28.07 ± 0.71	18.19 ± 1.14**	0.69 ± 0.21**
40% O2	24.02 ± 1.23*	11.41 ± 0.78**	0.12 ± 0.09**
Inhibition of photosynthesis (% kPa–1 increase in O2)	0.50 ± 0.23	1.55 ± 0.16**	2.90 ± 0.40**
Stomatal conductance, gs (mol H2O m−2 s−1)			
5% O2	0.39 ± 0.02	0.29 ± 0.06	0.31 ± 0.08
21% O2	0.32 ± 0.02*	0.24 ± 0.05*	0.28 ± 0.05*
40% O2	0.29 ± 0.02**	0.22 ± 0.04**	0.19 ± 0.03**
Transpiration rate, Tr (mmol H2O m−2 s−1)			
5% O2	8.56 ± 0.30	6.77 ± 1.29	7.25 ± 0.07
21% O2	6.08 ± 0.30**	4.53 ± 0.77*	4.78 ± 1.12*
40% O2	6.77 ± 0.37**	4.75 ± 0.07*	4.32 ± 0.46**
Internal/External CO2 concentration, Ci/Ca			
5% O2	0.64 ± 0.02	0.58 ± 0.06	0.93 ± 0.01**
21% O2	0.59 ± 0.03	0.60 ± 0.06	0.96 ± 0.01**
40% O2	0.61 ± 0.04	0.69 ± 0.05	0.97 ± 0.01**

Plants were grown at HC for 12 weeks and then subjected to step increases in O_2_ concentration for 60 min each, at the end of which measurements were taken (see Methods). Values are means (±SE) of at least three biological replicates. O_2_ inhibition of photosynthesis was calculated as described by Dai *et al.* (1996). Significant differences were determined using Student’s *t*-test: for the *PGLP*-RNAi lines the means are compared with that of the wild-type at the same O_2_ concentration; for the wild-type the means are compared with that at 5% O_2_ concentrations. *P<0.05, **P<0.01.

In the wild-type, when O_2_ was increased from 5% to 21%, *A* decreased slightly (–6.3% compared to the last time-point at 5%) but then stayed stable over the rest of the 60-min measurement period (28.07 ± 0.71 µmol CO_2_ m^−2^ s^−1^) (Fig. 4, Table 1). L3 also maintained stable *A* over 60 min but had a greater initial decrease (–23.8%) and a significantly lower value of *A* (18.19 ± 1.14) compared to that of the wild type. In L11 the rate of photosynthesis was already very low in 5% O_2_ and it dropped further after the change to 21% O_2_ (–39.6%), followed by a decline to 0.69 ± 0.2 after 60 min. Increasing O_2_ to 21% also resulted in decreases in *g*_*s*_ and *Tr* in the wild-type (but no effect on *C*_i_/*C*_a_), and these decreases were greater in the RNAi lines (Table 1). L11 again exhibited a significant increase in *C*_i_/*C*_a_.

Increasing the photorespiratory pressure to 40% O_2_ again resulted in an initial relatively small decrease in *A* in the wild type (–6.6% compared to the last value at 21% O_2_), and *A* further declined over the remainder of the 60-min measurement period (–8.4% compared to the first value at 40% O_2_) (Fig. 4, Table 1). Similar results were obtained with L3 but again the decreases were greater, with *A* initially at –21.7% of the last value at 21% O_2_ and then continuously declining by a further 20% by the end of the 60-min period. L11 again showed an initial decrease (–26.4% compared to the last value at 21% O_2_) and further declined over the remainder of the measurement period (–75.4% compared to the first value at 40% O_2_), and thus photosynthesis was almost completely inhibited (Fig. 4, Table 1).The inhibition of photosynthesis by O_2_ (Dai *et al.*, 1996) was consistently low in the wild-type (0.50 ± 0.23% kPa^–1^ increase in O_2_) and was significantly increased in the RNAi lines according to the degree of PGLP reduction: thus, L3 had greater inhibition (1.55 ± 0.16), followed by L11 (2.90 ± 0.40). Changes in *g*_*s*_ were congruent with those in *A* since it declined slightly in the wild-type and more distinctly in the RNAi lines. In contrast, *Tr* and *C*_i_/*C*_a_ were very similar to 21% O_2_ (Table 1). Taken together, these results suggested that C_4_ photosynthesis relies on optimal 2PG degradation through PGLP.

### Impairment of carbon utilization has a major impact on amino acid metabolism

**Fig. 5.**
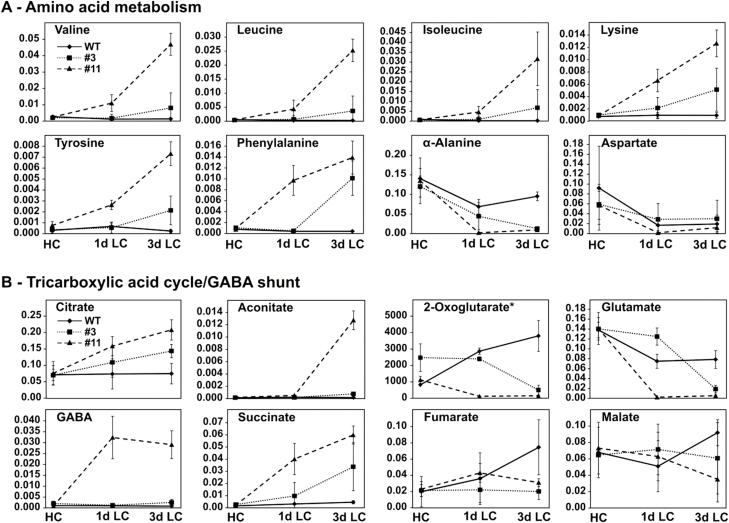
Levels of metabolites in *Flaveria bidentis PGLP*-RNAi lines L3 and L11 compared to the wild-type (WT) during transition from high to low CO_2_. Plants were grown for 12 weeks in high CO_2_ (HC, 1%) and then transferred to low CO_2_ (LC, atmospheric air) before being sampled at 1 d and 3 d. (A) Intermediates of amino acid metabolism and (B) intermediates of the TCA cycle. Values are means (±SD) (*n*>3). *All metabolite levels were determined using GC-MS and are expressed as relative amounts (arbitrary units mg^−1^ FW) except for 2-oxoglutarate, which was determined using LC-MS/MS and is expressed as absolute amounts (pmol mg^−1^ FW). For statistical evaluation see the Supplementary Datasheet S1.

Next, we examined how other branches of central carbon metabolism adapted to the impairment of carbon utilization. Most strikingly, following the transfer to LC the two transgenic lines accumulated high amounts of both branched-chain (valine, leucine, and isoleucine) and aromatic (tyrosine and phenylalanine) amino acids, which varied according to their reduction in PGLP (Fig. 5A, Supplementary Datasheet S1). All these amino acids tended to decrease in the wild-type. After 3 d in LC, the wild-type exhibited significantly lower amounts of valine (52%), isoleucine (30%), leucine (51%), tyrosine (78%), and phenylalanine (52%) compared to HC. In contrast, at the same time-point both transgenic lines had increases in valine (L3 ~3-fold; L11 ~17-fold), leucine (L3 ~7-fold; L11 ~46-fold), isoleucine (L3 ~8-fold; L11 ~36-fold), tyrosine (L3 ~7-fold; L11 ~23-fold), and phenylalanine (L3 ~13-fold; L11 ~18-fold). Similar effects were observed with lysine (L3 ~7-fold; L11 ~16-fold), which was unaffected in the wild-type, and threonine (L3 ~2-fold; L11 ~5-fold), which was decreased in the wild-type (20%). A major impact was also found for amino acids related to nitrogen fixation and the C_4_ cycle. By day 3 in LC, glutamate and alanine had decreased in the wild-type relative to HC (57% and 68%, respectively), and they had also decreased in the transgenic lines but to a greater extent (glutamate: L3 13%, L11 4%; alanine: L3 11%, L11 7%). The contents in HC mostly similar between genotypes (Fig. 5, Supplementary Datasheet S1). Aspartate also decreased to a similar extent in the wild-type (21%), L3 (51%), and L11 (21%) in response to the transfer in LC. This consistent pattern could also be seen in methionine and proline, with both gradually decreasing in a similar manner in all genotypes until day 3 in LC. Very little variation was observed for cysteine during acclimation to low CO_2_ (Supplementary Datasheet S1).

In addition to the changes observed in amino acids, we also examined sugars and polyols and found that several of them (e.g., sucrose, maltose, lactose, raffinose, and glycerol) showed generally similar patterns during the CO_2_ transition across all the genotypes (Supplementary Fig. S4A). Both the transgenic lines accumulated mannose (L3 ~2-fold; L11 ~4-fold) and xylose (L3 ~2-fold; L11 ~3-fold) in a similar way to that seen for lysine up to day 3 in LC, whereas mannitol was only slightly elevated in L11 (virtually PGLP-free). Minor and less consistent variations were found in fructose (significantly higher at day 3 in LC in both RNAi lines) and glucose (significantly higher at day 3 in LC in L11), with both sugars already showing some variations in HC (Supplementary Fig. S4A, Supplementary Datasheet S1).

### *PGLP*-RNAi lines exhibit a disrupted TCA cycle but only minor changes in the amounts of other organic acids

Several studies have provided evidence that the TCA cycle in C_3_ plants responds to impairments in photorespiration at the metabolic level (e.g. Obata *et al.*, 2016), and we therefore sought to determine whether the same might be true for the C_4_ species *F. bidentis*. In the wild-type, we found that most of the TCA-cycle and related metabolites analysed were not significantly altered by the CO_2_ transition (e.g. citrate, aconitate, GABA, and malate) (Fig. 5B); however, increases in 2-oxoglutarate, succinate, and fumarate were observed, with significantly higher levels being present on day 3 in LC. No significant differences were observed between the transgenic lines and the wild-type in HC (Fig. 5B, Supplementary Datasheet S1). After the shift to LC, both the transgenic lines accumulated citrate up to day 3 (L3 ~2-fold; L11 ~3-fold), while aconitate was only elevated on day 3 in LC in L11. Greater increases were observed for succinate (L3 ~7-fold; L11 ~13-fold) and GABA (L3 ~3-fold; L11 ~33-fold) on day 3 in LC. In contrast, decreased amounts of 2-oxoglutarate (L3 58%; L11 4%), fumarate (L3 27%; L11 41%), and malate (L3 66%; L11 38%) were observed compared to the wild-type at the same time-point (Fig. 5B). Apart from the changes in TCA-cycle intermediates, other organic acids (e.g. lactate, maleate, malonate, and hydroxyglutarate) did not show pronounced differences between the wild-type and the transgenic lines. We only found increased amounts of gluconate (L3 ~1.5-fold; L11 ~3.5-fold), and lower levels of quinic acid (L3 17%; L11 3%) and shikimate (L3 30%; L11 17%) after 3 d in LC (Supplementary Fig. S4B, Supplementary Datasheet S1).

## Discussion

Photorespiration is the key to maintaining high rates of C_3_ photosynthesis in the presence of O_2_ and has also been shown to be essential in organisms that exhibit carbon-concentration mechanisms (Suzuki *et al.*, 1989; Nakamura *et al.*, 2005; Eisenhut *et al.*, 2008*b*; Zelitch *et al.*, 2009; Timm *et al.*, 2012; Rademacher *et al.*, 2016); however, much less is known about photorespiration in C_4_ photosynthesis. Only the role of the peroxisomal enzyme glycolate oxidase (GOX) has been examined in transgenic maize (Zelitch *et al.*, 2009). Beyond its function in converting glycolate to glyoxylate during photorespiration, GOX also contributes to cellular H_2_O_2_ homeostasis and is required for other metabolic routes, such as the glyoxylate cycle. To specifically test whether photorespiration is essential in C_4_ plant species, we generated *Flaveria bidentis* lines that had reduced abundance of PGLP, the first, plastid-localized step of the pathway. PGLP is the only known enzyme that specifically degrades 2PG and is thus crucial for minimizing the carbon losses caused by the oxygenation of RuBP. In addition, 2PG has been shown to exhibit substantial regulatory potential with regard to the enzymes involved in carbon utilization both *in vitro* and *in vivo* (Anderson, 1971; Kelly and Latzko, 1976; Haimovich-Dayan *et al.*, 2015; Flügel *et al.*, 2017; Jiang *et al.*, 2018).

### *Flaveria bidentis PGLP*-RNAi lines are comparable to photorespiratory mutants of C_3_ plants, cyanobacteria, and green and red algae

The rate of photorespiration is considerably lower in C_4_ compared to C_3_ plants due to the enrichment of CO_2_ in the close vicinity of Rubisco (Hatch, 1971; Ogren, 1984; Laisk and Edwards, 1998; Sage, 2004). Consequently, it is widely assumed that carbon influx into photorespiration and its physiological significance in C_4_ organisms is minor. To test this hypothesis, we generated a set of eight *PGLP*-RNAi lines (Fig. 1; Supplementary Fig. 1) and comprehensively examined the two lines that displayed the lowest PGLP levels among the primary transformants (L3, ~3–5%; L11, ~0%; Fig. 1). The growth and phenotypic response of the transgenic lines in normal atmospheric air and during transition from high to low CO_2_ provided evidence that C_4_ photosynthesis in *F. bidentis* tolerated significant reductions in PGLP protein (Figs 1, 2, Supplementary Fig. S2). A clear photorespiratory phenotype was established only if PGLP protein abundance was below 5%, whereas similar symptoms in C_3_ Arabidopsis had already appear at a PGLP level of ~20% (Fig. 1; Flügel *et al.*, 2017). Considerable overlap in the phenotypic responses of the C_3_ and C_4_ model was observed at very low PGLP levels. C_4_*F. bidentis* exhibits stunted growth in 1% CO_2_ (Fig. 2), similar to the Arabidopsis *pglp1* mutants (Timm *et al.*, 2012; Flügel *et al.*, 2017). Photosynthesis in the *PGLP*-RNAi lines was also reduced at low O_2_ concentration (5%) (Fig. 4), which is again similar to Arabidopsis (e.g. measured at 2% O_2_, Somerville and Ogren, 1979). This result shows that 2PG is also detrimental to C_4_ photosynthesis and substantiates the idea that it is the most critical photorespiratory intermediate as well as a very potent inhibitor of carbon utilization. This might also explain why external sucrose application does not result in a full recovery of the *PGLP* mutant phenotype as observed for other photorespiratory mutants (Timm and Bauwe, 2013).

Overlap between the responses of both types of photosynthesis was also found in CO_2_ assimilation and the metabolic profile of the transgenic lines. We carried out experiments with rapid changes in the prevailing CO_2_/O_2_ ratios and hence in 2PG production (Figs 2–4). Similar to previous studies, *F. bidentis* wild-type plants maintained stable values of *A* when exposed to varying O_2_ concentrations (Fig. 4; Table 1), which is because C_4_ plants have strongly decreased oxygen inhibition compared to C_3_ plants (e.g. Ku *et al.*, 1991; Dai *et al.*, 1996; Maroco *et al.*, 1997, 1998). This further indicated that the wild-type could tolerate some fluctuations in 2PG, as long as its degradation through photorespiration operated efficiently. The reduction of photosynthesis in the transgenic lines was much greater. In addition to longer-term survival in atmospheric air (Fig. 2), L3 displayed lower values of *A* but could maintain stable CO_2_ fixation, at least under ambient O_2_ concentrations (Fig. 4). The significantly increased O_2_ inhibition of *A* in L3 (Table 1) suggested that it was not simply determined by the kinetic properties of Rubisco and the CO_2_ concentration but that it was also affected by the level of 2PG; however, the virtual absence of PGLP in L11 could not be tolerated. When PGLP protein abundance fell below ~3% of the wild-type level clearly increased the O_2_ sensitivity of *A* even at low O_2_ concentrations (Table 1) and caused the complete collapse of photosynthesis at higher O_2_ concentrations. The gradual decrease in *A* under 5% O_2_ also supports the assumption that photorespiration in C_4_ plants is lower but that its function is essential for maintaining carbon fixation, even at a low oxygen partial pressure. Similar observations have also been made during photosynthetic measurements of the maize *GOX* mutant, which also shows reduced *A* (~30–40%) at 1% O_2_ (Zelitch *et al.*, 2009). However, the inhibition of CO_2_ assimilation was much more severe in the *PGLP*-RNAi line 11, again confirming the strong inhibitory potential of 2PG, especially for RuBP regeneration. Mechanistically, decelerated RuBP regeneration is probably a consequence of inhibition of TPI and SBPase, as recently shown experimentally for Arabidopsis mutants with reduced PGLP activities (Flügel *et al.*, 2017).

In response to impaired carbon assimilation, both *PGLP*-RNAi lines exhibited severe stress symptoms that were very similar to those observed with C_3_ plants and cyanobacteria ([Bibr CIT0015][Bibr CIT0062]; Orf *et al.*, 2016). High amounts of branched-chain and aromatic amino acids accumulated in response to 2PG accumulation (Fig. 5A). These findings may have been due to impaired carbon utilization and the subsequent onset of carbon starvation involving protein degradation. Both lines showed some reprogramming of the TCA cycle and an induced GABA shunt (Fig. 5B). Several studies have shown that the TCA cycle in C_3_ plants responds to impairments of photorespiration on the metabolic level despite the fact that the underlying molecular mechanism remains unknown (e.g. Obata *et al.*, 2016). It is likely that the inhibition of photosynthesis, as well as the subsequent reduction of the carbon supply and distribution, induces enhanced protein degradation. Consequently, a higher proportion of free amino acids can be used as alternative respiratory substrates via the different flux modes of the TCA cycle (Sweetlove *et al.*, 2010; Araújo *et al.*, 2011) as a type of rescue programme. However, the general metabolic footprint of the *Flaveria bidentis PGLP*-RNAi lines was very similar to that observed with comparable Arabidopsis lines (Flügel *et al.*, 2017), although perhaps with a less pronounced intensity.

### C_4_ photosynthesis is more robust towards 2PG toxicity due to the distribution of specific enzymatic steps between mesophyll and bundle sheath cells

Upon transfer of the wild-type from high to low CO_2_, only 2PG reflected the ‘classic’ pattern of photorespiratory intermediates (low in HC, initial increase after transfer to LC, followed by normalization) in C_4_ species (Fig. 3). Thus, 2PG appears to be a reliable and conserved marker for the induction of photorespiration across various species (Eisenhut *et al.*, 2008*a*; Orf *et al.*, 2016; Flügel *et al.*, 2017). While most of the other photorespiratory intermediates were unchanged in the wild type, glycerate was markedly increased after the transfer from HC to LC (Fig. 3). This was probably because glycerate kinase, the final enzyme of the photorespiratory pathway, is located exclusively in the chloroplasts of mesophyll cells of NADP-ME C_4_ plants. The elevated amounts of glycerate under photorespiratory conditions could thus reflect its role as a transport metabolite (Usuda and Edwards, 1980; Weber and von Cammerer, 2010; Döring *et al.*, 2016), and the higher amounts may eventually be needed to drive metabolite diffusion between the mesophyll and the bundle sheath cells.

The transgenic lines strongly accumulated 2PG (Fig. 3, Supplementary Datasheet S1), whereas only minor changes were observed in other photorespiratory intermediates when compared to other pathway mutants (Timm *et al.*, 2012; Eisenhut *et al.*, 2017; Flügel *et al.*, 2017). The absolute amounts of 2PG were more than twice as high in the PGLP-deficient L11 when compared to the Arabidopsis *pglp1* knockout mutant (2667 versus 1112 pmol mg^−1^ FW) (Flügel *et al.*, 2017). Interestingly, the amount of 2PG in L3 (~1705 pmol mg^−1^ FW) was also considerably above that of *pglp1* and, in combination with its long-term survival in atmospheric air, suggests that *F. bidentis* is able to cope with much higher 2PG levels than the C_3_ plant, at least transiently. However, it can be hypothesized from the gas-exchange measurements (Fig. 4) that the CBC in the C_4_*PGLP*-RNAi lines was strongly impaired, which corresponds to the reported regulatory potential of 2PG on this pathway, particularly at the TPI and SBPase steps (Anderson, 1971; Flügel *et al.*, 2017). Inhibition of the CBC would obviously have a substantial impact on the allocation of triose phosphates from the chloroplast to the cytosol and, in addition, on the distribution of carbon between source and sink tissue (Fig. 2). Mechanistically, the malfunctioning of triose phosphate export could well be hampered due to the high amount of inorganic phosphate sequestered in 2PG and thus the impaired functioning of the triose phosphate translocator (Walters *et al.*, 2004). However, compared to C_3_ plants, the consequences of TPI inhibition could be less severe due to the presence of the 3PGA-triose phosphate shuttle between the mesophyll and bundle sheath chloroplasts in the C_4_*F. bidentis*. The reductive phase of the CBC takes place partly in the mesophyll cells of NADP-ME plants such as *F. bidentis* (Weber and von Cammerer, 2010; Gowik *et al.*, 2011). While 2PG exclusively accumulates in the bundle sheath chloroplasts and thus inhibits TPI in this location, 3PGA can be exported from the bundle sheath and reduced in the mesophyll (Weber and von Cammerer, 2010). This may allow the maintenance of a residual CBC with non-functional bundle sheath TPI and could provide a partial explanation for the higher 2PG resistance of C_4_ plants.

## Conclusions

The repression of *PGLP* in *F. bidentis* clearly demonstrated that photorespiration is essential for C_4_ species. This finding is consistent with an earlier study that demonstrated that a *GOX* knockout is lethal to maize (Zelitch *et al.*, 2009). *Flaveria bidentis* lines that were severely depleted in PGLP phenotypically resembled Arabidopsis *PGLP* mutants, indicating that the strong mutant symptoms are indeed caused by the toxic effect of photorespiratory metabolites, particularly 2PG. Interestingly, C_4_*F. bidentis* plant was still viable at much lower PGLP levels compared to C_3_ Arabidopsis. This was probably due to the operation of the C_4_ cycle itself, which reduces 2PG production. In addition, it appeared that *F. bidentis* could tolerate higher amounts of 2PG than Arabidopsis. This could be due to the division of labour between the mesophyll and bundle sheath cells with regards to the CBC, which warrants further studies of enzyme activities and their regulation by photorespiratory intermediates, particularly 2PG.

## Supplementary data

Supplementary data are available at *JXB* online.

Table S1. Oligonucleotides used during this study.

Fig. S1. Generation and verification of *Flaveria bidentis PGLP*-RNAi lines.

Fig. S2. PGLP amounts and phenotypes of selected *PGLP*-RNAi lines grown in soil in atmospheric air.

Fig. S3. PGLP amounts and phenotypes of selected *PGLP*-RNAi lines of the T2 generation grown in soil in atmospheric air.

Fig. S4. Metabolite levels in transgenic lines and the wild-type during the transition from high to low CO_2_.

Datasheet S1. Metabolite contents in the transgenic lines and the wild-type during the transition from high to low CO_2_.

Datasheet S2. Gas-exchange data at varying oxygen concentrations.

## Supplementary Material

Supplementary Figures S1-S4 and Table S1

Supplementary Data Sheet S1

Supplementary Data Sheet S2

## Abbreviations:

2PG: 2-Phosphoglycolate
3PGA: 3-phosphoglycerate
CBC: Calvin–Benson cycle
HC: high carbon
LC: low carbon
PGLP: phosphoglycolate phosphatase
RuBP: ribulose-1,5-bisphosphate
TPI: triosephosphate isomerase
SBPase: sedoheptulose-1,7-bisphosphatase.
