# Supplementary material for: Efficient 2-phosphoglycolate degradation is required to maintain carbon assimilation and allocation in the C4 plant Flaveria bidentis
Source: J Exp Bot. 2018 Oct 23;70(2):575–87. doi: 10.1093/jxb/ery370 (PMC6322630; doi:10.1093/jxb/ery370)
Supplement: Supplementary Figures S1-S4 and Table S1 [file ery370_suppl_supplemental_figures_s1-s4_and_table_s1.pdf]

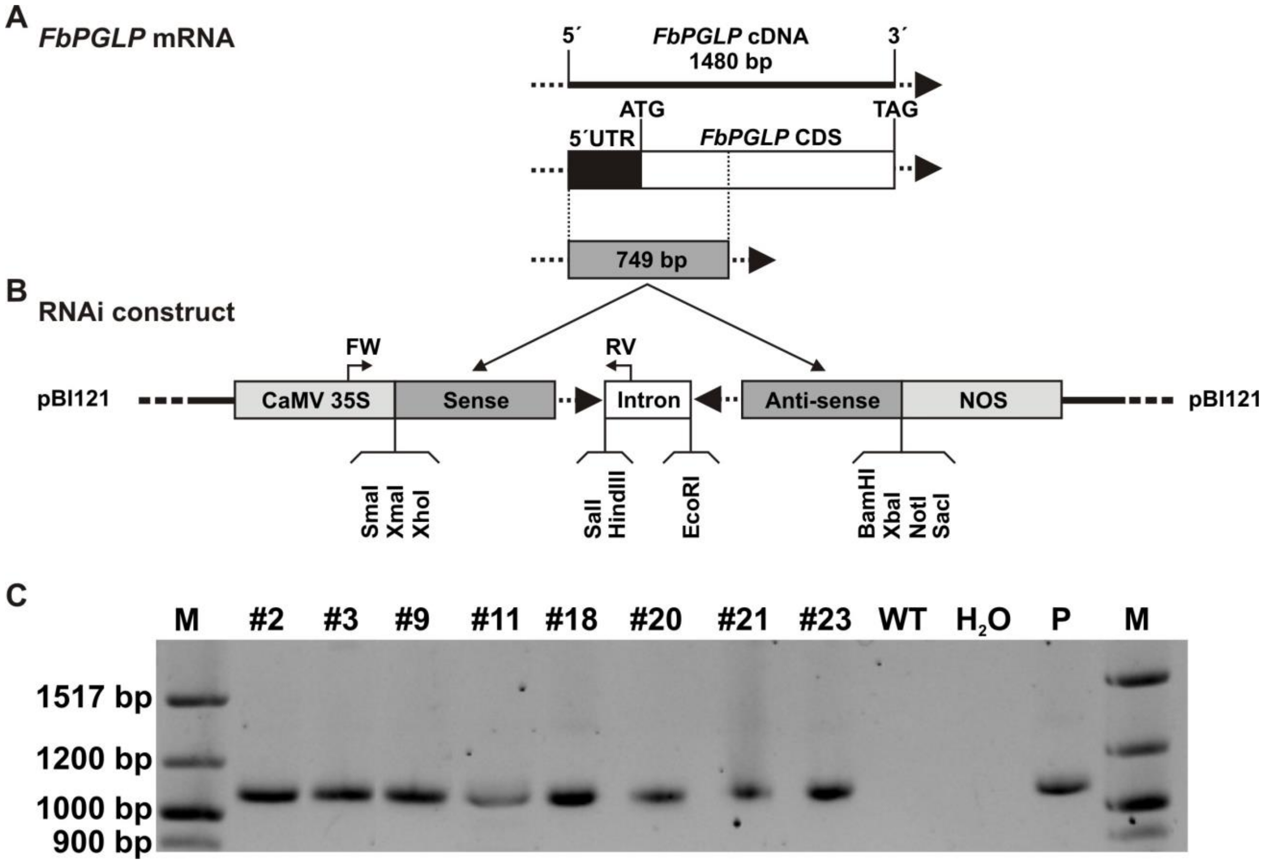

Supplemental Figure S1. Generation and verification of *Fb PGLP* RNAi lines.

(A) Sketch of the *Fb* cDNA encoding *PGLP* and the respective 749 bp sub-fragment used for the siRNA approach. **(B)** Structure of the pBI121 plasmid bearing the *PGLP* siRNA cassette (pBI121-*PGLP*-~~si~~RNAi). **(C)** PCR verification of eight selected transgenic lines (WT – wild type DNA control, H_2O - negative control, P – pBI121-*PGLP*-~~si~~RNAi plasmid control). Labels FW and RV indicate oligonucleotide binding sides used for PCR verification of the transgenic lines yielding a 1079 bp fragment.

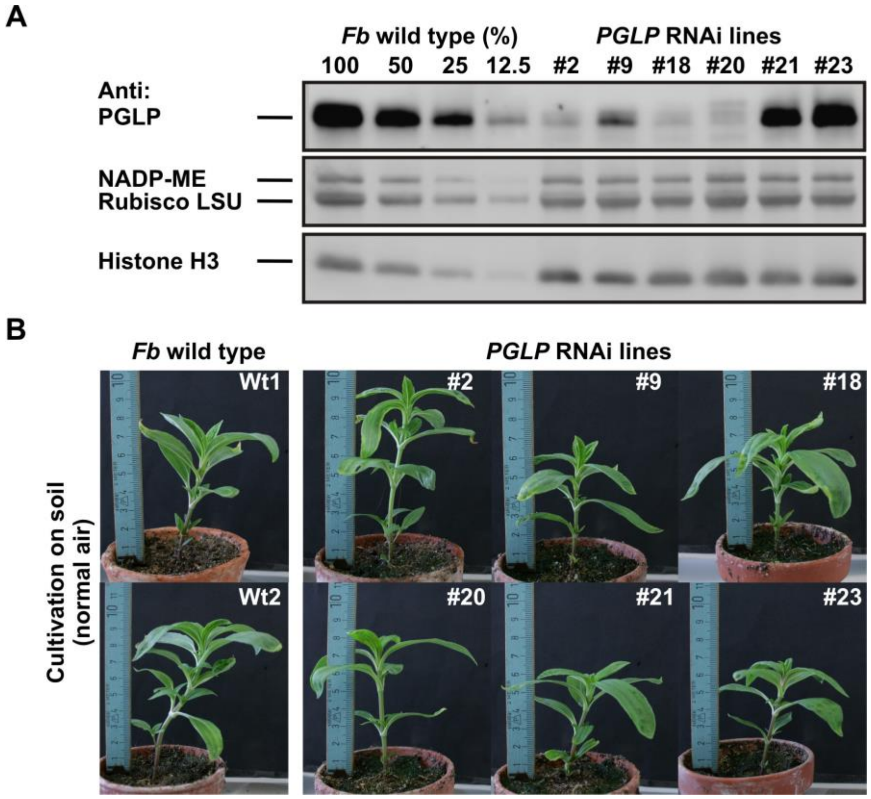

Supplemental Figure S2. PGLP amounts and Phenotype of selected *PGLP* RNAi lines grown in normal air on soil.

(A) Immunoblots of leaf-protein extracts (60 µg) of the wild type and *PGLP* ~~si~~RNAi lines using a PGLP specific antibody. To estimate PGLP amounts, signals of the transgenic lines were compared to a dilution series of the wild type protein extract. Signals of the NADP-ME, Rubisco LSU and histone H3 were used as loading control. Similar results were obtained during independent experiments. **(B)** Phenotype of wild type *Fb* and *PGLP* ~~si~~RNAi lines 2, 9, 18, 20, 21 and 23 in normal air grown on soil for 1 week.

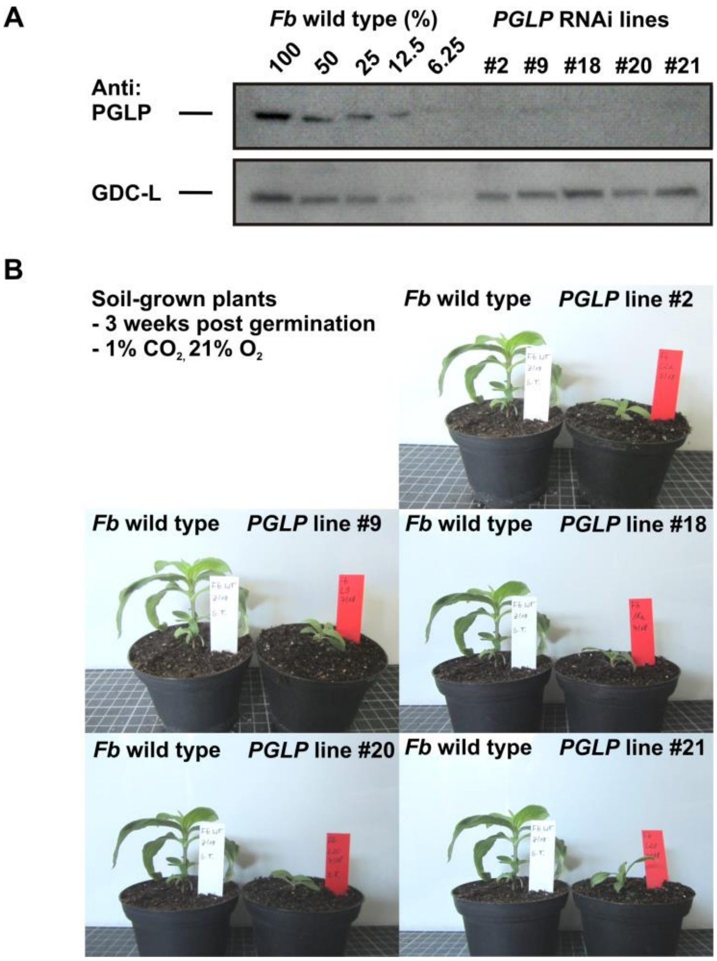

Supplemental Figure S3. PGLP amounts and Phenotype of selected *PGLP* RNAi lines (T2 generation) grown in normal air on soil.

(A) Immunoblots of leaf-protein extracts (10 µg) of the wild type and *PGLP* ~~si~~RNAi lines using a PGLP specific antibody. To estimate PGLP amounts, signals of the transgenic lines were compared to a dilution series of the wild type protein extract. Signals of glycine decarboxylase L-protein (GDC-L) were used as loading control. Similar results were obtained during independent experiments. **(B)** Phenotype of wild type *Fb* and *PGLP* ~~si~~RNAi lines 2, 9, 18, 20 and 21 in normal air grown on soil for 2 weeks.

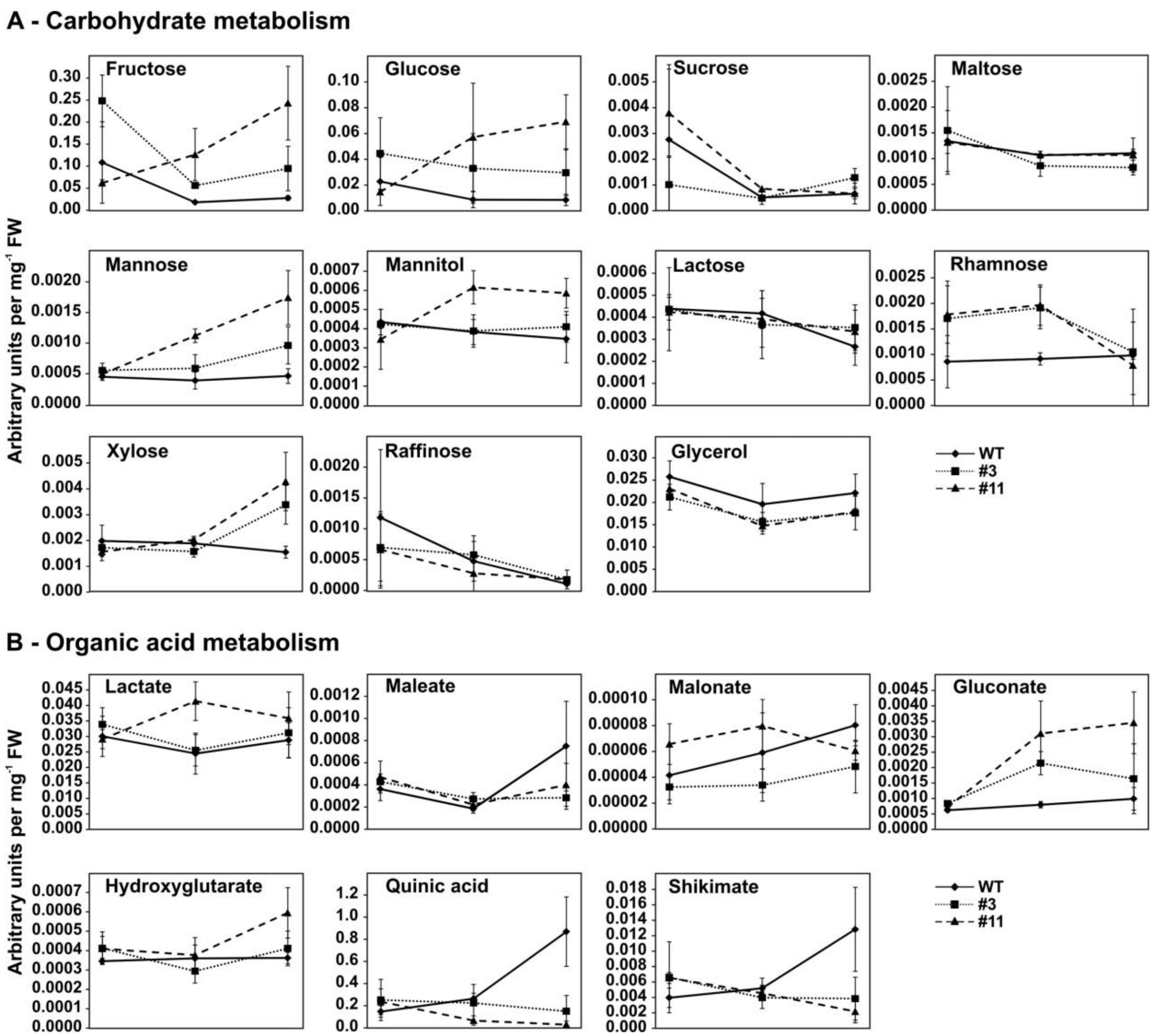

Supplemental Figure S4. Metabolite levels in transgenic lines and the wild type during CO_2 transition.

For metabolite analysis leaf material was harvested (source leaves, fourth fully expanded leaf pair) during the CO_2 transition experiment in HC and after 1 and 3 days in LC (Fig. 2). Shown are mean values ± SD (N>3) from intermediates of **(A)** carbohydrate and **(B)** organic acid metabolism. Metabolites were determined by GC-MS (arbitrary units [AU] per mg^{-1} FW). For statistical evaluation see full numerical dataset provided as Supp. Datasheet S1.

Supplemental Table S1. Oligonucleotides used during this study.

**Primer**	**Sequence (5´to 3´)**
PGLP-RNAi-sense-fw	ATA**AAGCTT**TCCACCATCTTCCGGACCCCCAA
PGLP-RNAi-sense-rev	ATA**CTCGAG**GGTACACAAACCACCTTGAACCAT TGG
PGLP-RNAi-antisense-fw	ATA**GAATTC**TCCACCATCTTCCGGACCCCCAA
PGLP-RNAi-antisense-rev	ATA**GGATCC**GGTACACAAACCACCTTGAACCATTGG
A-fw	ATA GGC GCG CCC GTC GAC GGG TAC CAT TTA AAT GCG ATC GCC CCG GGC TCG AGA TA
B-rev	TAT CTC GAG CCC GGG GCG ATC GCA TTT AAA TGG TAC CCG TCG ACG GGC GCG CCT AT
35S-FW	TGCAAACACACAAGACAATGGAC
Act11-Int-RV	CCAACCACGTCTTCAAAGCAAG
